# Giant serpentine aneurysm of the internal cerebral artery and mandibular aneurysm: a case report

**DOI:** 10.1186/s41016-019-0175-6

**Published:** 2019-11-21

**Authors:** Qiao Deng, Wen Feng Feng

**Affiliations:** Department of Neurosurgery, Nanfang Hospital of Southern Medical University, Guangzhou, 510515 China

**Keywords:** Giant serpentine aneurysm, Tubridge flow diverter, LEO stent, Mandibular aneurysm

## Abstract

**Background:**

Giant serpentine aneurysms (GSA) originate from saccular or spindle aneurysm, dissimilar from dissected aneurysm, that are defined as partially thrombosed giant aneurysms with tortuous internal vascular channel. The clinical and neuroradiologic characteristics are clarified and the mechanism of formation and the efficacy of double stent implantation in GSA are discussed.

**Case presentation:**

An 18-year-old man presented himself with a GSA arising from the internal cerebral artery (ICA). In addition, a mandibular aneurysm (MA) arose from the external cerebral artery (ECA). Success was achieved in treating GSA through endovascular treatment with double stents implanted in the parent artery, which were LEO stent and Tubridge flow diverter. After 1 year of follow-up, three-dimensional reconstruction of blood vessels revealed the disappearance of the serpentine access of GSA, which was found to be replaced with a roughly normal vascular structure.

**Conclusions:**

Double stent implantation has provided a feasible treatment option for giant serpentine internal carotid aneurysms and eliminated the possibility of causing collateral circulation occlusion. Therefore, it represents a simple and suitable treatment method for anatomical structure and operation.

## Background

Serpentine aneurysm of the internal carotid artery is usually known as a giant aneurysm (> 25 mm in diameter) with residual tortuous vascular channels, which are the specific intra thrombotic passageways rather than the residual lumens of the parent artery [1]. These abnormal channels are made up of non-endothelial blood vessels losing normal elastic thin layer or medium, either central or eccentric, and the blood flow in the channels is slower than in the entrance. These non-endothelial thrombotic internal channels allow blood flow to the distal branch of the parent artery and ensure supply to the crucial areas in the brain parenchyma. The mechanism of GSA formation is yet to be fully understood, and it is speculated to be associated with the repeated intramural bleeding and thrombosis in initial fusiform aneurysms. GSA contains a thick fibrous wall and peripheral calcified thrombus, which generally exhibits progressive mass effects (midline shift and perifocal brain tissue edema on computed tomography). In addition, dissection would initiate the process of GSA formation. The mass effect and progressive neurologic deficits caused by GSA in the internal carotid artery were discovered not to be as significant as in the middle cerebral artery.

## Case presentation

**Fig. 1:**
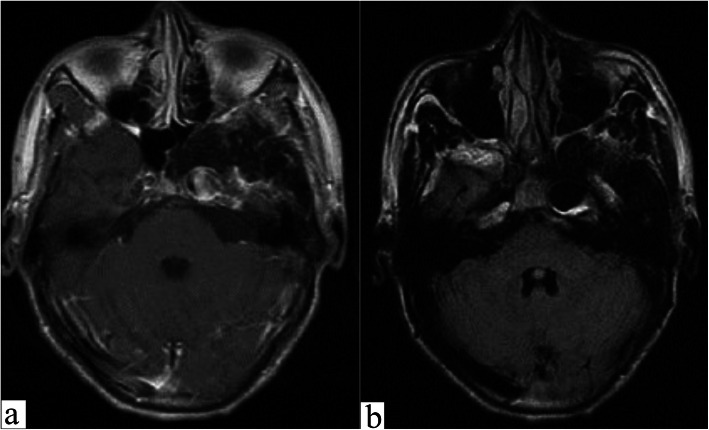
Magnetic resonance imaging shows an aneurysm of the left internal carotid artery. **a** Enhanced scanning shows mild uneven enhancement and heterogeneous signal intensities representing variable stages of thrombosis. **b** T2 magnetic resonance imaging shows aneurysm is low signal and blood vessel wall calcification

**Fig. 2:**
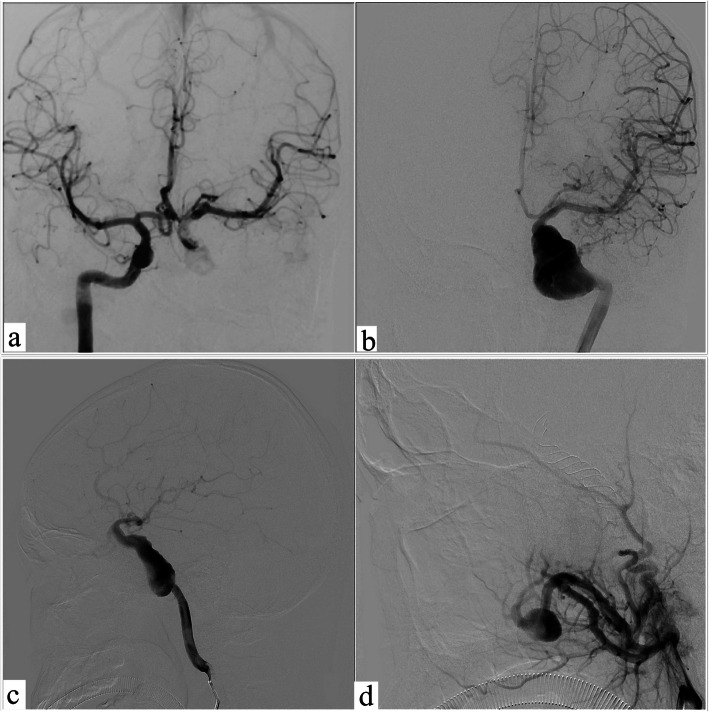
The left internal and external carotid angiography. **a** Balloon occlusion test showed patency of anterior communicating artery. **b–c** Digital subtraction angiography anteroposterior and lateral views display the separate inflow and outflow channels of the giant serpentine aneurysm; Weakening of contrast medium filling at the distal end of the channel. **d** Left external carotid artery angiography suggests intramaxillary aneurysms

An 18-year-old man was admitted to our department showing the symptoms of blurriness. A 1-year history was reported on blurriness and visual indistinctness that had progressively worsened over the past 2 weeks. He has a record of tuberculosis and takes anti-TB drugs on a regular basis. Neurologic examinations at the time of admission revealed no focal neurologic deficits except for oculomotor paralysis. Magnetic resonance imaging (MRI) indicated that the diameter of intracranial segment in the left internal carotid artery was significantly dilated, the inner diameter was approximately 14 mm, the left anterior cerebral artery, and the starting point of the MCA were slightly dilated as well, and the structure of the left cavernous sinus area lacked clarity. There was a circular liquid signal shadow spotted under the inner wall of the left maxillary sinus, the diameter of which was about 16 mm (Fig. 1). Further cerebral angiography indicated that the left internal carotid artery GSA was about 48.61 mm × 23.40 mm in size, 4.65 mm in diameter of proximal vessels, and 3.16 mm in diameter of distal vessels (Fig. 2).

**Fig. 3:**
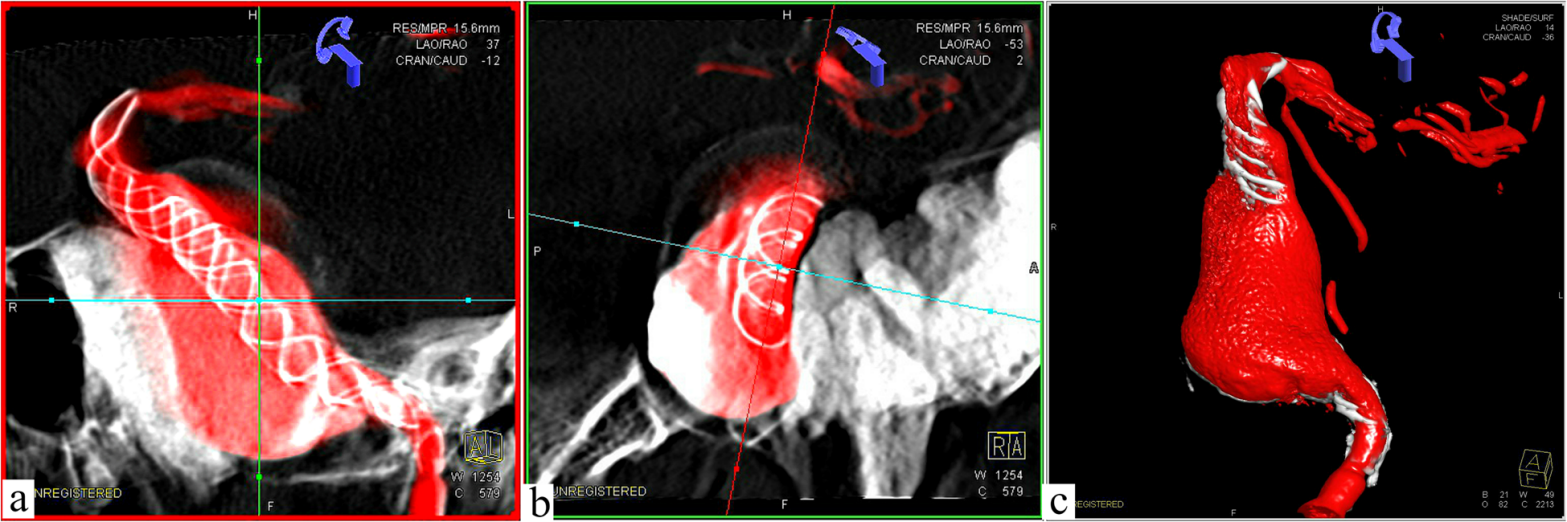
Three-dimensional vascular reconstruction to show the morphology of stents. **a** The overlap of the middle part of the stents is clearly visible. **b** The end of the bracket is stretched and anchored. **c** Specific structure of stents and GSA

**Fig. 4:**
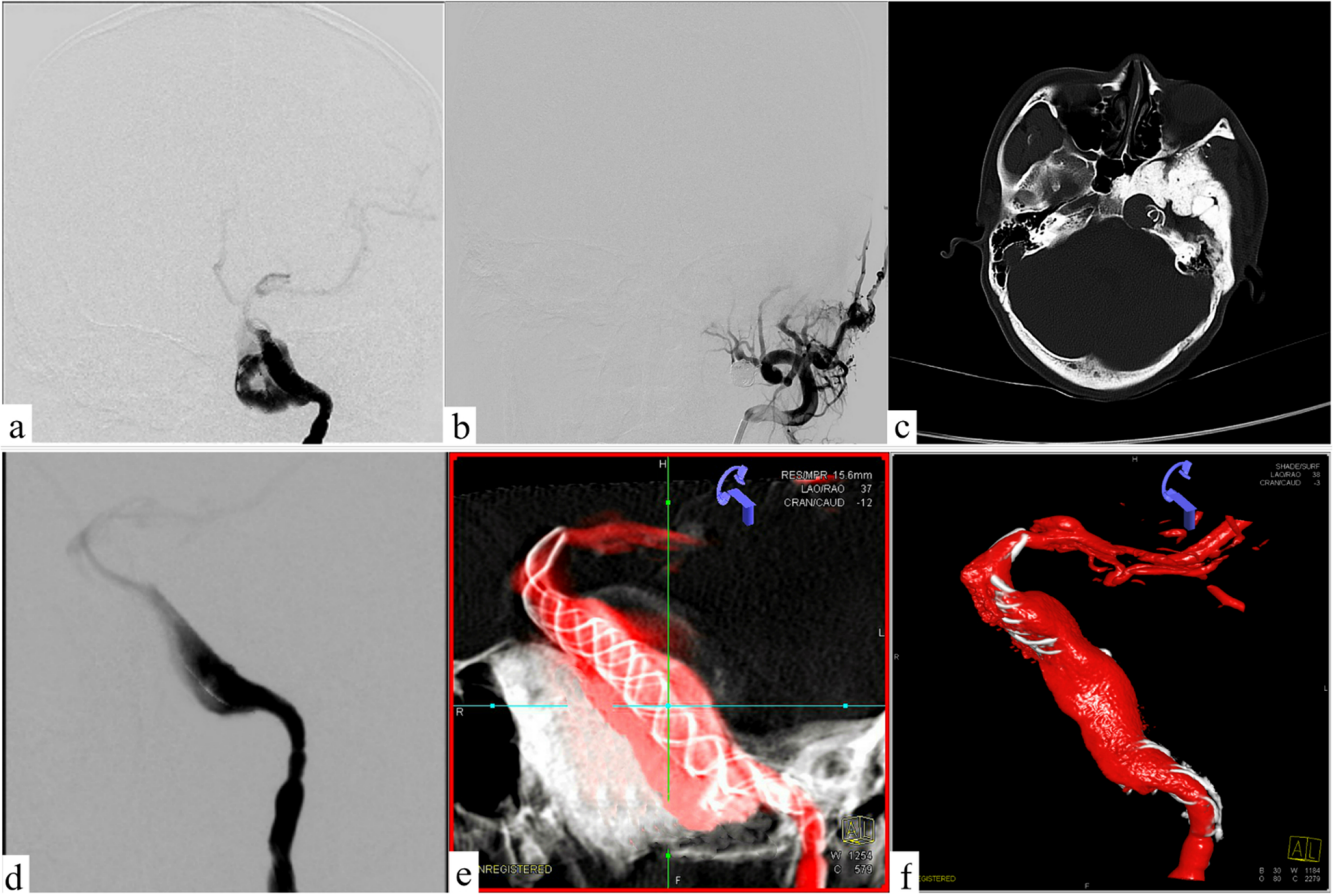
Postoperative follow-up of CT and DSA. **a** Left internal carotid artery angiography shows the redirection of the aneurysm inflow jet. **b** External carotid arteriography shows complete occlusion of MA. **c** Postoperative reexamination of CT. **d** DSA reveals the variation of flow direction in the stents. **e**–**f** 3D-DSA display the transform volume and morphology of GSA

His family was informed of these conditions and signed a consent form prior to the operation. The balloon occlusion test conducted previous to surgery indicated that the anterior communicating artery was open and the contralateral internal carotid artery had a good compensatory blood supply, as a result of which direct trapping of the parent artery was taken as an alternative method. However, ipsilateral MA has a potential of further growth which could lead to paralysis of facial nerve compression, which is due to higher blood flow to the external carotid artery. His family members rejected this traumatic treatment method and thus the insertion of stents was chosen to reconstruct the original structure of the vessel. Therefore, the following surgery was performed: antiplatelet therapy (100 mg aspirin +  75 mg clopidogrel daily) was carried out at least 3 days prior to the operation, and he underwent surgery after being diagnosed with anesthesia. Firstly, a LEO (6.5 mm × 75 mm) stent was put through an Echelon-10 catheter located in the MCA, and then the stent was released at a slow pace and completely through the GSA. Meanwhile, the head was anchored at the distal end of the parent artery. Subsequently, the trans 300 floppy guidewire was switched to the marksman on the MCA. The Tubridge flow diverter (6.5 mm × 45 mm) was easily placed into the designated position where it was attached to the LEO stent that was well anchored before. With the assistance of X-ray, the Tubridge flow diverter was slowly released to overlap with the LEO stent wall and the aneurysm neck was completely covered. Ultimately, digital subtraction angiography (DSA) revealed obvious retention of contrast agent in GSA, and three-dimensional vascular reconstruction demonstrated that the walls of the parent artery were reconstructed by both Tubridge and LEO stent (Fig. 3). Then, the guide catheter to the external carotid artery was replaced, liquid glue (50%, 1.5 ml) was injected, and three coils were released through the microcatheter. Finally, the right femoral artery with an 8F Angio-Seal vascular occlude was blocked. The patient needed to avoid stent thrombosis by taking aspirin for a long period of time, although the thrombo-elastography (TEG) test indicated that arachidonic acid inhibition rate (AA%) and inhibition rate on ADP receptor of clopidogrel (ADP%) were within the normal range. After a follow-up observation for 1 year, DSA revealed that the aneurysms were almost completely occluded and the abnormal serpentine channels disappeared. Meanwhile, the MA disappeared as well. The two stents overlapped in the parent artery with excellent adhesion, and no stenosis or displacement was discovered. Three-dimensional revascularization indicated a significant reduction in aneurysm volume, and the mass effect was reduced as shown by CT scan (Fig. 4).

## Discussion and conclusions

GSAs reportedly account for approximately < 0.1% of all kinds of intracranial aneurysms and approximately 17.6% of giant serpentine aneurysms which can transform into the former [2]. At present, there remain no specific studies on epidemiology and pathogenesis in this respect. GSA originating from the MCA or its branch vessels is almost common in half of the cases, the internal carotid artery in 13%, and the anterior cerebral artery in less than 3%. GSA can be associated with recurrent intramural bleeding and thrombosis in the initial fusiform aneurysm. Senbokuya et al. reported a case of a fusiform aneurysm in the anterior cerebral artery that transformed into GSA within a space of 5 months with intramural thrombus [3]. GSA usually exhibits progressive mass effects due to its 1–4-mm-thick fibrous wall and peripheral calcified thrombus (midline shift and perifocal brain tissue edema on CT scan). Nevertheless, subarachnoid hemorrhage is not regarded as a common presentation of GSAs [4]. Thus, GSA progression can be characterized by progressive mass effects, rupture bleeding, and spontaneous occlusion. Among them, the development of mass effect is identified as the most common in MCA [5].

The major clinical symptoms of GSA include headache, seizures, hemiplegia, facial paralysis, cranial nerve palsy, and visual impairment. Sari et al. described a case of GSA where acute complete thrombosis was manifested after the first diagnostic angiography, with no sign of neurological impairment spotted. Radiological revealed persistent thrombotic aneurysms with peri-wall calcification after a 3-year follow-up observation [6]. McLaughlin et al. reported a case of GSA that was spontaneously completely thrombosed, and the distal territory of the occluded MCA branch was irrigated by numerous collaterals or unnamed arteries [7]. However, Mahadevan et al. reported a case of GSA that led to a rapid process of neurologic deterioration (Hemiplegia and speech impairment) after occlusion, due to a poor collateral circulation [8].

Cerebrovascular angiography represents the most commonly used standard for the diagnosis of GSA, which is capable to clearly demonstrate the characteristic serpentine channels, offer support for normal distal circulation of the maternal artery, and provide a reference for subsequent treatment [9]. CT angiography and MRI can be performed to screen for GSA and perfusion CT can clearly indicate the ischemic changes occurring in brain tissue around aneurysms. In this patient, MRI clearly demonstrates the mass effect, perivascular enhancement, and hypointense flow void representing the patent channel within the aneurysm [10].

A rare case of both GSA and MA is described, and it is speculated that congenital vascular wall dysplasia and weakness could contribute to the formation of GSA and MA. The large snake-like channel completely fuses the opening of the anterior choroid artery and the posterior communication artery, and these fixed perforation blood flow ensures blood supply to the significant functional areas in the brain. Retrograde blood flow in the serpentine channel results in the reduction of blood supply to the distal arteries, as manifested in a noticeable decline of contrast agent filling in the ipsilateral anterior and middle cerebral arteries [11]. In addition, retrograde blood flow is suspected to increase blood pressure in the external carotid artery through the high-flow bypass, thus further promoting the growth of MA [12]. The MA between the internal maxillary artery and the facial artery is possibly more floating and changeable than any other portions of the ECA, and the regrowth of the MA with GSA has the potential to cause delayed complications [13, 14].

The placement of a double stent reconstructs the direction of blood flow in the channel. Therefore, it can be expected that the reduction of perfusion pressure in the terminal artery is less severe with the serpentine channel inlet [15]. The turbulence of blood flow resulting from the abrupt change in the arterial diameter is conducive to thrombus formation, which may result in occlusion of important perforating vessels, hemiplegia, and visual impairment that may not be apparent previously. Therefore, flow diverter (FD) slightly shorter than the longest diameter of GSA was selected and released below the expected opening of the anterior choroid artery to avoid perforating artery occlusion caused by high metal coverage. After follow-up observation, overlapping of FD we could be performed selectively based on the actual progress of GSA.

GSA therapy is designed for a complete elimination of aneurysm cavities, reduction of mass effects, and reconstruction of distal circulation. A combination of its large size, lack of aneurysmal neck, and dependence on distal vessels presents certain technical challenges to the treatment. GSA is supposed to be treated possibly soon, as there is a possibility that normal blood vessels subsequently merge into the aneurysm cavity, thus causing difficulty in revascularization and leading to progressive neurological deterioration, spontaneous thrombosis, or intracranial hemorrhage. The latest FD including Pipeline, Tubridge, and Surpass provide a better option for the treatment of GSA [16, 17]. FD can change the direction of hemodynamics by means of intracavitary reconstruction of the parent artery and dismissing the serpentine channel. It allows the indirect exclusion of the sac from the vasculature and facilitates the growth of endothelial cells and neointimal tissue across the aneurysm neck. The controlled clinical trial was designed to assess the safety and efficacy of the Tubridge flow diverter in the treatment of large or giant aneurysms compared with enterprise stent-assisted coiling, and the results of 6-month follow-up imaging included complete occlusion rates of 75.34% for the Tubridge [18]. At present, there remain no studies focusing on the complete occlusion rate of GSA following FD implantation. Therefore, a LEO stent combined with Tubridge was chosen to implant for this patient. With consideration given to the serpentine channel, uncertain anchoring site, and the stability and tolerance of the stent, a decision was taken to abandon the double-pipeline or Tubridge bridging technology. The first release of LEO stent contributed to initially establishing the release path of subsequent supports and provided sufficient radial support. Then, the Tubridge was released to overlap with LEO, thus increasing the metal coverage density of the aneurysm neck and achieving the reconstruction of the parent artery. The patient’s vision returned to normal without the occurrence of any new neurological defects. DSA revealed that abnormal serpentine channel basically disappeared, and almost complete occlusion was achieved.

## Conclusions

In this report, a description is made of a partially thrombosed serpentine aneurysm and mandibular aneurysm that arising from the left ICA and ECA, which was successfully treated by endovascular surgery with favorable prognosis. GSA usually originates from large spherical aneurysms, which prompts the speculation that this patient’s circular mandibular aneurysm, if left untreated, has a greater likelihood of developing GSA in the next few years. Therapeutic options for GSAs are dependent on the manifestation of the aneurysm, anatomical location, and the features exhibited by the feeding and draining vessels for distal tissue. In this report, it is suggested that double stent implantation provides a safe and effective way to treat GSA. Besides, it removes the need for direct surgical clipping or embolization that may result in disastrous consequences.

## Data Availability

Not applicable.

## Abbreviations

CT: Computed tomography
DSA: Digital subtraction angiography
ECA: External carotid artery
FD: Flow diverter
GSA: Giant serpentine aneurysm
ICA: Internal carotid artery
MA: Mandibular aneurysm
MCA: Middle cerebral artery
MRI: Magnetic resonance imaging
